# Clinicopathological and Prognostic Value of Survivin Expression in Surgically Resected Pancreatic Ductal Adenocarcinoma

**DOI:** 10.3390/cancers14143494

**Published:** 2022-07-18

**Authors:** Christian Vay, Shahrooz Babaei, Sami-Alexander Safi, Levent Dizdar, Alexander Rehders, Lena Haeberle, Christoph Roderburg, Sven H. Loosen, Irene Esposito, Wolfram T. Knoefel, Andreas Krieg

**Affiliations:** 1 Department of Surgery (A), University Hospital Duesseldorf, Heinrich-Heine-University, Moorenstrasse 5, 40225 Duesseldorf, Germany; christian.vay@med.uni-duesseldorf.de (C.V.); shahrooz.babaei@med.uni-duesseldorf.de (S.B.); sami-alexander.safi@med.uni-duesseldorf.de (S.-A.S.); levent.dizdar@med.uni-duesseldorf.de (L.D.); rehders@med.uni-duesseldorf.de (A.R.); knoefel@med.uni-duesseldorf.de (W.T.K.); 2 Institute of Pathology, University Hospital Duesseldorf, Heinrich-Heine-University, Moorenstrasse 5, 40225 Duesseldorf, Germany; lenajulia.haeberle@med.uni-duesseldorf.de (L.H.); irene.esposito@med.uni-duesseldorf.de (I.E.); 3 Clinic for Gastroenterology, Hepatology and Infectious Diseases, University Hospital Duesseldorf, Heinrich-Heine-University, Moorenstrasse 5, 40225 Duesseldorf, Germany; christoph.roderburg@med.uni-duesseldorf.de (C.R.); sven.loosen@med.uni-duesseldorf.de (S.H.L.)

**Keywords:** survivin, BIRC5, inhibitor of apoptosis protein family, IAP, ductal adenocarcinoma of the pancreas, PDAC, tissue microarray, TMA

## Abstract

**Simple Summary:**

In spite of recent optimisation of surgical therapy and multimodal treatment options, late diagnosis and devastating overall prognosis continue to characterise pancreatic cancer. The inhibitor of apoptosis protein survivin has been established as a relevant if not unambiguously understood factor in the formation and progression of pancreatic ductal adenocarcinoma (PDAC) being upregulated already in the early stages of tumourigenesis. Therefore, we analysed the expression of survivin in primary PDAC and lymph node metastases in an ample collective of 236 patients and demonstrated that cytoplasmic as nuclear overexpression of the protein correlated significantly with clinicopathological indicators of disease progression and, accordingly, showed prognostic relevance. The findings support the use of survivin as a biomarker to further explore the aggressiveness of PDAC subtypes and encourage its therapeutic approach as a molecular target to expand current chances for disease survival and potential cure.

**Abstract:**

Background: Survival after surgery for pancreatic ductal adenocarcinoma (PDAC) remains poor. Thus, novel therapeutic concepts focus on the development of targeted therapies. In this context, inhibitor of apoptosis protein (IAP) survivin is regarded as a promising oncotherapeutic target. However, its expression and prognostic value in different tumour compartments of PDAC have not been studied. Methods: Immunohistochemical analysis of survivin in different PDAC tumour compartments from 236 consecutive patients was correlated with clinicopathological variables and survival. Results: In comparison to healthy pancreatic tissue high nuclear (*p* < 0.001) and high cytoplasmic (*p* < 0.01) survivin expression became evident in the tumour centre, along the invasion front and in lymph node metastases. Cytoplasmic overexpression of survivin in tumour centres was related to the presence of distant metastasis (*p* = 0.016) and UICC III/IV stages (*p* = 0.009), while high cytoplasmic expression at the invasion front grouped with venous infiltration (*p* = 0.022). Increased nuclear survivin along the invasion front correlated with perineural invasion (*p* = 0.035). High nuclear survivin in tumour centres represented an independent prognostic factor for overall survival of pancreatic tail carcinomas (HR 13.5 95%CI (1.4–129.7)) and correlated with a limited disease-free survival in PDAC (HR 1.80 95%CI (1.04–3.12)). Conclusion: Survivin is associated with advanced disease stages and poor prognosis. Therefore, survivin will help to identify patients with aggressive tumour phenotypes that could benefit from the inclusion in clinical trials incorporating survivin inhibitors in PDAC.

## 1. Introduction

With a persistently rising incidence, pancreatic ductal adenocarcinoma (PDAC) is currently the seventh leading cause of malignoma-related death and is estimated to become the second leading cause of fatalities associated with cancer in 2030 [1,2]. Termed as one of the “recalcitrant” cancers by US legislation [3], PDAC overall 5-year survival rates remain poor ranging from 5–10% with 80–85% of the patients diagnosed in an advanced or metastatic stage of the disease [4,5]. Further, surgical resection supplemented by an adjuvant chemotherapy remains the only curative therapy approach, whereby only 20% of the patients with localised PDAC reach postoperative 5-year survival [6].

Poor patient outcome in mainly asymptomatic pancreatic cancer is related to late diagnosis at advanced tumour stages and high rates of cancer recurrence even after complete resection (R0) with curative intent including the assessment of the circumferential resection margin (CRM) [7]. Despite acknowledgeable advances in patient outcome, more sophisticated biomarkers are urgently needed for earlier PDAC detection in patients at risk. If such markers equally serve as targets for disease-specific therapy approaches, their clinical implementation should lead to novel treatment options, finally improving disease prognosis.

Heterogeneous genomic alterations in pancreatic cancer comprising numerous mutations of tumour suppressor genes (inter alia KRAS, CDKN2A, CDK4, TP53, STK11, ATM, MLH1, MSH2, MSH6, PALB2, SMAD4/DPC4, BRCA1, and BRCA2), and their role in PDAC carcinogenesis and progression have been broadly investigated and described [6,8,9]. Aside from KRAS, TP53, and SMAD4 mutations, which are found in more than 50–90% of PDAC genomes [10], the remainder of the tumour suppressor genes mentioned here are neither commonly altered in the majority of the cases nor specific for the cancer entity itself [11,12]. Recently, immune checkpoint inhibitors have demonstrated surprising survival benefits in a broad spectrum of malignancies, but immunomodulatory interventions were found to be of limited success in PDAC, which was accredited to the immunosuppressive microenvironment [13,14]. However, molecular stratification of PDAC subtypes has improved therapy response prediction discriminating patient subgroups due to prognostic indicators after adjuvant therapy [15,16,17].

While surgery remains the only chance of cure in pancreatic cancer, novel therapeutic concepts focus on the development of targeted therapies that specifically inhibit aberrant molecular pathways involved in cell proliferation and cell survival on the genomic, transcriptional, and proteomic levels in cancer cells to improve the survival perspectives of PDAC patients beyond operative treatment and existing chemotherapeutic or immunomodulatory treatment. The current lack of more effective compounds reflects the incomplete understanding of the molecular mechanisms responsible for the particular resistance and recurrence of PDAC. So far, targeted molecular and immunotherapeutic approaches, which have brought revolutionary therapy successes to the treatment of other solid malignancies, could not significantly improve the treatment of PDAC patients. To overcome the current therapeutic deficits, ongoing research aims to further decipher the opaque microenvironment of the pancreatic tumour, the high genetic instability of the cancer and, increasingly important, the immune microenvironment of PDAC [18].

In the course of malignant progression, pancreatic neoplastic cells acquire resistance mechanisms in impairing the initiation of apoptosis by the death ligands TNFα, FasL, and TRAIL, by the upregulation of Bcl-family proteins, and the overexpression of caspase inhibitors as the inhibitor of apoptosis protein (IAP) family members including survivin [19]. Additionally, in PDAC, survivin has been established as a relevant if not unambiguously understood factor in pancreatic cancer formation and progression that appears to be upregulated already in early stages of tumourigenesis [20].

Survivin, one of the eight members of the inhibitor of apoptosis family of multifunctional proteins with their innate anti-apoptotic effects on cell proliferation and migration, represents a promising target to improve antitumour therapy due to its upregulation and overexpression in a wide range of solid malignancies. While practically absent in healthy adult tissue, only proliferating cells during embryonal development and very few terminally differentiated, non-neoplastic tissues display survivin expression. Therefore, survivin complies with essential criteria for targeted tumour therapies and, hence, should minimize toxicity to physiological cell populations [21]. Intriguingly, therapeutics addressing survivin as a target have not been successfully introduced to clinical treatment for several reasons [22].

Encoded by the Baculoviral IAP Repeat Containing 5 gene (BIRC5) located at chromosome 17q25, survivin has proven its oncogenic and metastatic potential as well as its prognostic relevance in a broad variety of epithelial and endocrine malignancies. Among the eight members of the inhibitor of apoptosis (IAP) family of multifunctional proteins with their shared anti-apoptotic effects on cell proliferation and migration, survivin (BIRC5) is a highly conserved, unique protein delivering its anti-apoptotic and mitotic effects in the cytoplasm as well as in mitochondria and nuclei. By interception of its sister protein X-linked inhibitor of apoptosis protein XIAP (BIRC4) in the cytoplasm, survivin disrupts the activation of apoptosis by targeting caspase-3 and caspase-9 of the intrinsic pathway. In addition, this XIAP–survivin complex switches off caspase-8 in the death-ligand-dependent extrinsic pathway. Additionally, attaching to the aurora B kinase, survivin adds to the formation of the chromosomal passenger complex by appending the kinetochore to the microtubule securing the correct alignment and separation of chromosomes during mitosis. Both survivin and XIAP mediate the nuclear translocation of Nuclear Factor kappaB (NF-κB) supporting tumour cell invasion and metastasis [23]. A prevalent mechanism, by which activated NF-κB induces chemoresistance in PDAC, is an increased expression of cellular inhibitors of apoptosis including survivin and survivin has demonstrated a potential to predict chemotherapy response [19,24].

The purpose of the present study was to specifically analyse the expression of survivin in different tumour cell compartments of PDAC (e.g., tumour centre and invasion front) and corresponding regional lymph node metastases in a sufficiently high number of consecutively resected tumours by comparison to the physiological presence of survivin in adjacent non-neoplastic tissue of the pancreas. In a second step, the results were statistically correlated with clinicopathological parameters and post-operative patient survival data to assess the potential therapeutic and prognostic relevance of survivin in the treatment of PDAC in accordance with the “Reporting Recommendations for Tumour Marker Prognostic Studies (REMARK)” [25].

## 2. Materials and Methods

### 2.1. Patients

In total, 279 patients who had undergone surgery for ductal adenocarcinoma of the pancreas with curative intent at Duesseldorf University Hospital between 2003 and 2018 were screened for inclusion in the present study. Exclusion criteria were preoperative neoadjuvant therapy, macroscopically incomplete resection (R2), 30-day hospital mortality, pancreatic malignancies other than PDAC, and tissue samples with unsuitable tumour material. TNM staging including grading (G) and perineural tumour invasion (Pn) as well as lymphatic (L) and venous (V) vessel invasion were obtained from the original pathological reports and adapted to the 8^th^ edition of the Union Internationale Contre le Cancer (Union for International Cancer Control, UICC) TNM classification of malignant tumours [26]. Clinicopathological data including tumour relapse, metachronous metastasis, overall survival, disease-free survival, patient sex, and age at the time of surgery were collected and reviewed. The study was carried out in accordance with Good Clinical Practice, the Declaration of Helsinki after approval by the local ethics committee at the medical faculty of the Heinrich-Heine-University Duesseldorf (study number: 3821).

### 2.2. Tissue Microarray and Immunohistochemistry

Formalin fixed paraffin-embedded tissue blocks were retrieved from the Institute of Pathology at Duesseldorf University Hospital. Tissue microarrays (TMA) of paraffin-embedded tissues were constructed for this study each containing three representative tissue cores from the primary tumours (two derived from central tumour parts and one selected from the invasion front), two tissue samples of lymph node metastases, and one tissue sample of healthy pancreatic tissue if available for each patient. Accordingly, up to six cylinders of 1.0 mm in diameter were taken from their respective donor blocks and placed in paraffin recipient blocks with 0.5 mm distance between the cylinders. To visualise the protein expression of survivin by immunohistochemical staining (IHC), TMAs were cut into slides with a thickness of 2 µm. The staining was performed using the ZytoChem Plus HRP-DAB Kit (Zytomed Systems, Berlin, Germany) as described previously [27]. Briefly, after deparaffinisation and rehydration antigen unmasking was performed at >95 °C for 30 min in a microwave oven using a 3% trisodium citrate dihydrate buffer equilibrated at pH 6.0 for epitope retrieval, followed by cooling of the slides to room temperature. After rinsing in PBS with 0.1% Tween-20 (Sigma-Aldrich, St. Louis, MO, USA) for 5 min, endogenous peroxidase was inactivated by incubating the slides in 3% H_2_O_2_ in phosphate-buffered saline (PBS, pH 7.4) for 10 min at room temperature. Sections were then rinsed three times for 2 min in PBS with 0.1% Tween-20 before blocking unspecific protein binding sites using treatment solution provided with the kit for 10 min. After washing in PBS with 0.1% Tween-20 for 2 min, immunostaining was performed for 60 min at room temperature with polyclonal rabbit anti-survivin antibody (NB500-201; dilution 1:750; Novus Biologicals, Littleton, CO, USA). Isotype control was conducted using a rabbit immunoglobulin fraction (Code X0903; dilution 1:1000; Dako, Glostrup, Denmark). After three washing steps in PBS with 0.1% Tween-20 each for 2 min, the slides were incubated with biotinylated secondary antibody for 15 min. Three more washing steps of 2 min each in PBS with 0.1% Tween-20 followed, before the slides were covered with streptavidin-conjugated horseradish peroxidase (HRP) for 30 min. After 5 min of rinsing in distilled water, epitope-specific visualisation was achieved by incubating the slides with 2 drops of high contrast 3,30-diaminobenzidine (DAB). After rinsing in distilled water for 5 min, brief nuclear counter-staining with Mayer’s haemalaun was performed for 15 sec before the slides were dehydrated after washing in distilled water for another 5 min and finally covered with a rapid mounting medium (Entellan, Sigma-Aldrich, St. Louis, MO, USA).

For each immunohistochemical staining run, a tissue slide of pretested human colon and renal cell carcinoma known to express survivin intensively served as positive controls. For TMA analysis, survivin staining intensity and percentage of stained cells were scored by three independent investigators (S.B., S.-A.S., and L.H.) according to the immunoreactivity score (IRS) suggested by Remmele and Stegner with slight modifications: intensity was graded as absent 0 = no staining; 1 = weak staining; 2 = strong staining; 3 = very strong staining) as well as the percentage of positive cells (0 = no positive cells; 1 = <10% positive cells; 2 = 11–50% positive cells; 3 = 51–80% positive cells; 4 = 81–100% positive cells [28]). The product of the two attributes equalled the IRS ranging from 0 to 12.

### 2.3. Statistical Analysis

Differences of survivin expression levels in pancreatic cancer specimens, lymph node metastases, and adjacent non-neoplastic tissues were analysed using the Wilcoxon test. For numerical data, a correlation between clinicopathological variables and expression levels of survivin were examined using the Mann-Whitney U test. The Chi-square test was used for categorical data. For some analyses immunoreactivity scores were categorized in high (IRS > 4) and low (IRS ≤ 4) expression of survivin. The cut-off value for this categorisation was set according to the median IRS for survivin expression in all investigated primary tumour samples and lymph node metastases. Outcome measures included overall survival defined as the period from the date of surgery until the date of last follow-up or death of any cause. If applicable, disease-free survival was defined as the time from the date of surgery until the date of diagnosed metastases or local recurrence. Cases with incomplete tumour resection or patients who died within 30 days after operation were excluded from the survival analyses. Kaplan-Meier curves were generated and assessed using the log-rank (Mantel Cox) test and hazard ratios (HR) with 95% confidence intervals (95% CI) were calculated. For multivariate survival analysis, all variables were included into a cox regression analysis with forward likelihood ratio (LR) settings. Analyses were performed using SPSS statistics for Windows (version 25.0; SPSS, Inc., Chicago, IL, USA). A *p*-value < 0.05 was considered to indicate a statistically significant difference.

## 3. Results

### 3.1. Patient Characteristics and Outcome

From a total number of 466 surgically explored patients with PDAC, 136 patients who received gastroenteric bypass surgery as a palliative approach and 51 patients who succumbed to postoperative mortality within 30 days after surgery were excluded from the study. The remaining 279 patients who underwent oncological pancreatic resection for PDAC between 2003 and 2018 were enrolled in our study. From 22 patients, no paraffin-embedded tissues were available for research purposes from the archives of the Institute of Pathology at Duesseldorf University Hospital. After pathological re-evaluation of the remaining 257 patients by board-certified pathologists (L.H., I.E.), 21 cases had to be withdrawn due to insufficient tumour material for further immunohistochemical evaluation. Thereupon, 12 tissue-microarray (TMA) paraffin blocks were constructed comprising a maximum number of samples from 236 different patients and their corresponding tissue specimens. The clinicopathological characteristics of the patients enrolled are summarised in Table 1. While tumour grading (G) could be assessed in 235 patients of the cohort (99.6%), histopathological data on the infiltration of perineural sheaths (Pn), lymphatic vessels (L), and blood vessels (V) were available in 50.4%, 70.3%, and 69.9% of the cases, respectively.

The median age of patients at the time of surgery was 68 years (range 41–95 years). All patients met the predefined inclusion criteria for survival analysis. In 217 patients the primary tumour was located in the pancreatic head (91.9%), while in 19 patients the primary tumour was situated in the tail (8.1%). For the entire patient cohort, the follow-up period ranged from 1 to 180 months, during which 199 patients died (84.3%). The median overall survival of PDAC patients was 22.2 months (range 1–168 months). Of the 236 cases included in the study, for 94 patients (39.8%) detailed follow up information could be obtained to delimit disease-free survival from overall survival, which was calculated for the patient to a mean duration of 17 months (range 3–154 months).

### 3.2. Survivin Expression in Pancreatic Ductal Adenocarcinoma

Nuclear and cytoplasmic survivin expression could be investigated in 220 (93.2%) and in 189 (80.8%) of the 236 patients, respectively, both in the central and marginal (invasion front) areas of the tumours. In 148 (76.3%) of 194 patients with lymph node metastases (82.2%), tissue material was accessible for IHC analysis. Of the 236 cases, for 175 patients (74.2%), non-neoplastic pancreatic tissue was available for immunohistochemical evaluation. In 166 samples from the 236 patients (70.3%), survivin expression in the primary tumours could be directly compared to the staining results of adjacent non-neoplastic pancreatic tissue.

**Figure 1:**
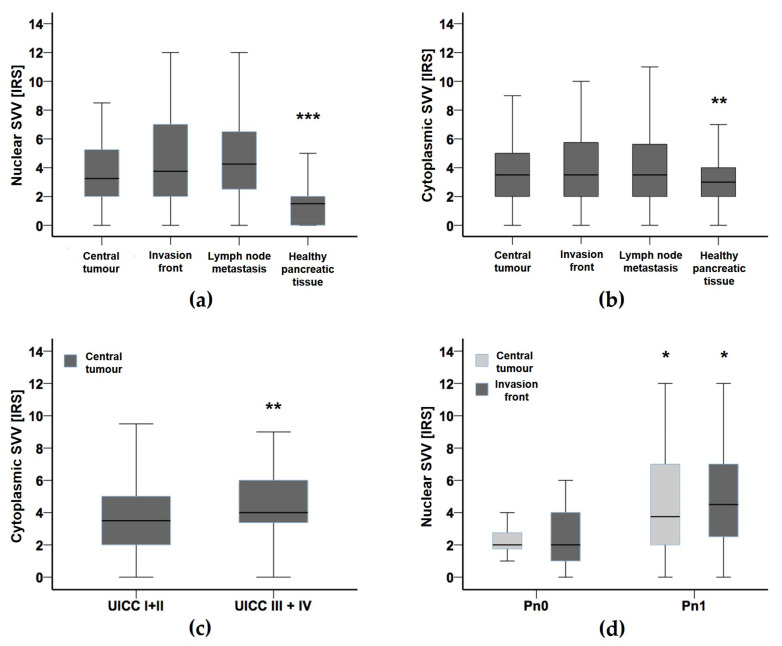
(**a**) Boxplot of nuclear survivin immunoreactivity expression in various tissue compartments of pancreatic ductal adenocarcinoma (PDAC): central tumour areas, tumour invasion front, lymph node metastasis, and healthy pancreatic tissue. Boxes depict the data sets comprised within the lower and upper quartiles with the median as bold cross-lines; whiskers welt data variability outside the upper and lower quartiles. *** indicates a *p* value ≤ 0.001 as calculated by Wilcoxon’s test. (SVV—survivin; IRS—immunoreactivity score). (**b**) Boxplot of cytoplasmic survivin expression in various tissue compartments of PDAC: central tumour areas, invasive margins, lymph node metastases, and healthy pancreatic tissue. ** indicates a *p* value ≤ 0.01 as calculated by Wilcoxon’s test (SVV—survivin; IRS—immunoreactivity score). (**c**) Boxplot of cytoplasmic survivin expression in central PDAC in correlation with low UICC stages I+II vs. high UICC stages III+IV. ** indicates a *p* value ≤ 0.01 as calculated by Mann-Whitney U test. (SVV—survivin; IRS—immunoreactivity score; UICC I+II/III+IV—grouped tumour stage classifications according to the Union Internationale Contre le Cancer (Union for International Cancer Control)). (**d**) Boxplot of nuclear survivin expression in central PDAC in correlation with perineural invasion Pn0 vs. Pn1. * indicates a *p* value ≤ 0.05 as calculated by Mann-Whitney U test. (SVV—survivin; IRS—immunoreactivity score).

**Figure 2:**
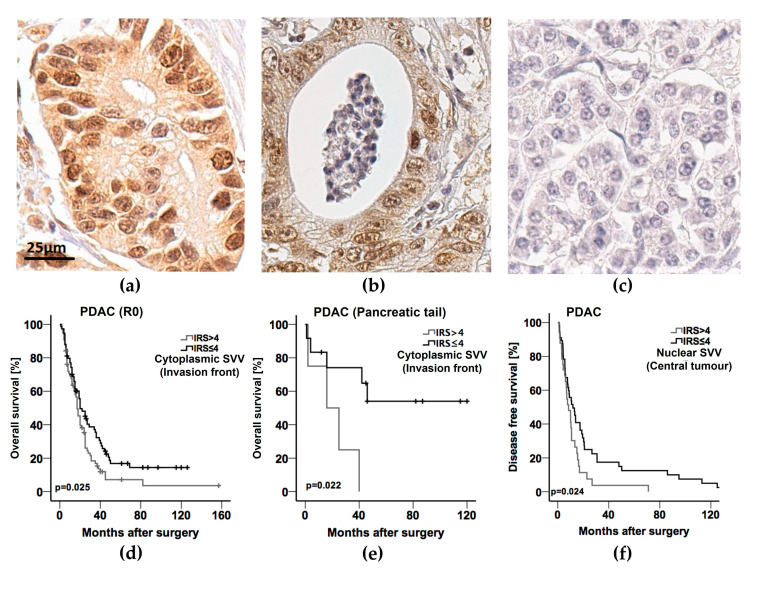
(**a**) Representative image of pancreatic ductal adenocarcinoma (PDAC) with an immunoreactivity score (IRS) of 4/12 for survivin expression in the central area of the tumour sample at 400× magnification. Scale bar indicates 25 µm. (**b**) Representative image of a PDAC lymph node metastasis with an IRS of 4/12 for survivin expression at 400× magnification. (**c**) Representative image of non-neoplastic pancreatic tissue with an IRS of 0/12 negative for survivin expression at 400× magnification. (**d**) Kaplan–Meier curve for overall survival of all R0 resected patients with PDAC of the pancreatic head and tail in correlation with cytoplasmic survivin expression at the invasion front (*p* = 0.025 as calculated by log rank test (SVV—survivin; IRS—immunoreactivity score). (**e**) Kaplan–Meier curve for overall survival of all patients with PDAC of the pancreatic tail in correlation with cytoplasmic survivin expression at the invasion front (*p* = 0.022 as calculated by log rank test) (SVV—survivin; IRS—immunoreactivity score); (**f**) Kaplan–Meier curve for disease-free survival of all patients with PDAC in correlation with nuclear survivin expression in central tumour areas (*p* = 0.024 as calculated by log rank test) (SVV—survivin; IRS—immunoreactivity score).

While the median immunoreactivity score (IRS) in non-malignant pancreatic tissue ranged as low as 1.0, the median IRS for nuclear survivin expression in central tumour areas amounted to 3.4, rising along the infiltrative tumour margins (invasion front) to 4.0, and was determined in lymph node metastases to 4.5, respectively (*p* < 0.001) (Figure 1a and Figure 2a–c).

In view of cytoplasmic survivin expression, the median IRS consistently averaged 3.5 in the central and peripheral tumour areas as well as in lymph node metastases, whereas in healthy pancreatic tissue the median cytoplasmic IRS for survivin was gradually lower with 3.0 (*p* < 0.01) (Figure 1b and Figure 2a–c).

Therefore, the cut-off values for high (IRS > 4) against low (IRS ≤ 4) survivin expression in the various tissue compartments were calculated from the median IRS values determined in the central and marginal tumour areas.

Starting statistical correlation between nuclear and cytoplasmic survivin expression levels and grouped clinicopathological variables, at first, the IRS scores of the various spatial compartments were compared to each parameter (Table 2 and Table 3).

Regarding nuclear survivin expression, high IRS scores (IRS > 4) at the invasion front were found to be significantly associated with perineural invasion (Pn1) (*p* = 0.035) and were observed more frequently in patients with distant metastasis (M1) (*p* = 0.067). High nuclear survivin scores in regional lymph node metastases occurred more often in PDAC patients with perineural invasion (Pn1) without reaching statistical significance though (*p* = 0.064).

On consideration of cytoplasmic survivin expression, high IRS scores (IRS>4) in the central tumour areas correlated significantly with advanced UICC stages (UICC III–IV) (*p* = 0.009) (Figure 1c) as well as with synchronous liver metastasis (M1) (*p* = 0.016). In a similar manner but with less statistical power, the majority of the patients with UICC stages III–IV tended to share high expression scores also at their invasion fronts (*p* = 0.084). By contrast, venous vessel invasion (V1) correlated with low IRS levels (≤4) (*p* = 0.022) along the tumour margins of the PDAC assessed.

### 3.3. Survival Analysis

Meeting the predefined inclusion criteria, all 236 PDAC patients could be subjected to univariate survival analysis: log-rank tests and Kaplan-Meier curves demonstrated that comparatively high age at the time of operation, the presence of distant synchronous liver metastases (M1), venous invasion (V1), and residual tumour cells after surgery (R1) were significantly associated with poor overall survival (Table 4). Apart from these correlations, only weak differentiation (G3) (*p* = 0.071) and strong cytoplasmic survivin expression along the tumour invasion fronts (IRS > 4) (*p* = 0.072) showed a marginal association with limited patient overall survival. In contrast, nuclear or cytoplasmic survivin expression levels in all other tissue compartments studied did not exhibit any prognostic relation to postoperative survival in the univariate analyses.

Multivariate Cox-regression analyses narrowed down high age at the time of operation, venous infiltration (V1), and microscopic tumour residuals (R1) as independent prognostic factors for overall survival (Table 4).

Prognostic subgroup analyses were performed for patients clustered according to the following characteristics (Table 5): (1) R0-resected PDAC independent of the primary tumour site, (2) R0-resected PDAC without distant metastasis (M0), (3) PDAC without distant metastasis (M0) including R1-resected patients, (4) PDAC of the pancreatic head, and (5) PDAC located in the pancreatic tail.

Patient age at the time of operation above the median of 68 years significantly correlated with a shortened postoperative overall survival in the subgroups of PDAC patients without postoperative residual tumour burden (R0), in the sub-categories of R0-resected patients without distant metastasis (M0) (*p* = 0.005), in the group of patients with distant metastasis (M1) independent of their R-status (*p* = 0.002), and in patients with PDAC of the pancreatic head (*p* = 0.024). The presence of distant metastasis (M1) was a negative predictive marker for overall survival in PDAC of the pancreatic head (*p* = 0.001) and, though without statistical significance, the pancreatic tail (*p* = 0.074). Moreover, shorter postoperative survival was marginally associated with the presence of regional lymph node metastasis (*p* = 0.074) in the latter cohort, while lymphatic vessel invasion (L1) reached statistical significance on the subject (*p* = 0.046). In PDAC of the pancreatic head, venous infiltration (V1) presented with a decreased overall survival (*p* = 0.046). The presence of residual tumour cells after resection (R1) was related to worse outcomes for patients with tumours of the pancreatic head (*p* = 0.056) and of the pancreatic tail (*p* = 0.047).

As summarized in Table 5, high cytoplasmic expression levels of survivin (IRS > 4) at the invasion front significantly correlated with limited overall survival in the subgroups of R0-resected patients (*p* = 0.025) (Figure 2d), of R0-resected patients without distant metastasis (both R0 and M0) (*p* = 0.029), of patients without metastasis (M0) independent of a residual tumour burden after PDAC resection (*p* = 0.037), and patients with tumours of the pancreatic tail (*p* = 0.022) (Figure 2e). Regarding overall survival, cytoplasmic survivin expression did not show statistical relevance for the prognostic outcome of the study cohort selected for patients with PDAC of the pancreatic head. An influence of nuclear survivin expression on postoperative survival was not derived from the outcome data of the explored subgroups of patients.

Multivariate subgroup analysis (Table 6) identified age above the median of 68 years as an independent marker for limited prognosis (overall survival) in patients without residual tumour and distant metastasis (both R0 and M0) (HR 1.58; 95% CI (1.032–2.231); *p* = 0.034) as well as in patients without metastasis (M0) independent of residual tumour (both R0 and R1) after resection (HR 1.5; 95% CI (1.1–2.1); *p* = 0.023). The presence of distant metastasis (M1) in patients with PDAC of the pancreatic head was identified as another independent variable predicting shortened postoperative overall survival (HR 2.06; 95% CI (1.3–3.3); *p* = 0.002).

Investigating the expression levels of survivin for their independent prognostic relevance, solely high expression scores (IRS > 4) in PDAC of the pancreatic tail were calculated to have a significant relation to overall survival in 19 patients of this small subgroup (HR 13.5; 95% CI (1.4–129.8); *p* = 0.024).

At last, survival analysis was performed also for disease-free survival available for a subset of 94 from 236 patients from the study cohort (39.8%) (Table 7). Univariate analysis identified present residual tumour (R1) (*p* = 0.043) and high nuclear survivin expression (IRS > 4) in the central (*p* = 0.024) as well as marginal (*p* = 0.050) tumour areas to significantly correlate with poor disease-free survival. Of these, only residual tumour burden (HR 1.885; 95% CI (1.065–3.161); *p* = 0.029) (R1) and high nuclear survivin expression (IRS > 4) in the central PDAC tissue areas (HR 1.798; 95% CI (1.037–3.118); *p* = 0.012) were designated as independent prognostic factors in the multivariate analysis (Table 7, Figure 2f).

## 4. Discussion

With an ever increasing incidence year on year [5], PDAC remains one of the few tumour entities sharing an extremely poor prognosis with an overall 5-year survival rate of 5–10% [4]. While surgical resection and adjuvant chemotherapy remain indispensable for curative treatment, targeted therapies have been failing to improve patient outcome and long-term survival markedly and, therefore, could not be successfully introduced to the clinical setting of PDAC treatment so far [6].

In accordance with its key role in cell cycle progression, apoptosis suppression, and cell migration and due to its frequent expression in a wide spectrum of epithelial neoplasia, the inhibitor of apoptosis protein (IAP) family member survivin (BIRC5) has been established as a marker for disease progression and prognostic outcome in several solid malignancies representing a promising and inventive target for molecular therapy approaches [29,30,31,32,33,34,35,36,37,38,39,40,41].

Even though several studies have attributed cancer characteristics and poor patient outcome to survivin overexpression in pancreatic cancer cell formations and apoptotic pathway deregulation as an established mechanism of carcinogenesis of this entity [19,23,42,43,44], the protein’s role in the formation and progression of PDAC remains to be investigated more profoundly.

Both its cytoplasmic and nuclear expression have been attributed to the suggested oncogenic functions of survivin as (1) an apoptotic inhibitor and (2) a mitotic effector with inconsistent and, in part, opposing correlations. However, the distinct functions of survivin in its two balanced intracellular storages—cytoplasm and nucleus—remain to be fully understood. While the nuclear pool is thought primarily to enhance cell proliferation, cytoplasmic survivin apparently has its major role as a cytoprotective player in controlling cell survival [45,46]. Further investigations of the underlying functional mechanisms and corresponding pathways of subcellular distribution are needed to resolve the exact roles of cytoplasmic and nuclear survivin and its isoforms in the survival and proliferation of malignant cells.

Therefore, we investigated the expression patterns and the prognostic role of cytoplasmic and nuclear players in central and marginal areas as well as in corresponding regional lymph node metastases from 236 PDAC patients who underwent surgical resection with curative intent. The findings were correlated with clinicopathological parameters and postoperative survival to determine the potential of survivin as a marker for disease progression and prognosis.

In comparison to the basal level of survivin protein expression in adjacent healthy, non-neoplastic tissue of the pancreas, survivin expression was significantly higher in the central and marginal parts of the primary tumours and in secondary lymph node metastases. Whereas the mean cytoplasmic survivin expression rates appeared relatively levelled, nuclear expression scores revealed a gradual increase from central tumour areas to the invasion front, and, beyond this, to regional lymph node metastases apparently mirroring PDAC progression.

Distinguishing cytoplasmic from otherwise nuclear expression, Sarela et al. described that of 52 pancreatic adenocarcinomas investigated 88% presented with cytoplasmic localisation of survivin, whereby strong expression significantly correlated with increased cellular proliferation as measured by Ki-67 co-expression, and, surprisingly, increased apoptosis determined by TUNEL assays [42]. Before, Satoh et al. assessed survivin occurrence in pancreatic carcinoma cell lines and various pancreatic tissue types by immunohistochemistry, immunoblotting, and reverse transcription-polymerase chain reaction (RT-PCR) to examine the interrelation of survivin expression with tumour apoptosis and tumourigenesis. While survivin was not detected in samples of healthy pancreatic tissue (0%) and chronic pancreatitis (0%), intraductal papillary mucinous neoplasia (IPMN) (56.3%) and PDAC (76.9%) exhibited rising percentages of preponderantly cytoplasmic survivin in a course from normal and inflammatory conditions to pre-malignant and malignant pancreatic lesions suggesting an upregulation of survivin early in tumourigenesis [20]. In this study, high survivin expression levels in the adenocarcinomas correlated significantly with a reduction of tumour cell apoptosis.

Our statistical correlations revealed that high cytoplasmic IRS scores in the central tumour areas and—to a lesser degree—high cytoplasmic survivin presence along the invasion front correlated with advanced UICC stages also linking elevated survivin levels in the tumour cells towards systemic disease progression. Consistently, patients with synchronous liver metastasis (M1) significantly more frequently shared an overexpression of cytoplasmic survivin in the central regions of the corresponding primary lesions than cases without distant metastases (M0). Interestingly, venous vessel infiltration (V1) correlated with a down-regulation of cytoplasmic survivin at the invasive margins of primary PDAC.

Nuclear overexpression of survivin at the tumour invasion front was significantly associated with perineural invasion (Pn1) and occurred comparably often in patients with distant metastasis (M1). In regional lymph node metastases, again, elevated nuclear survivin levels were more frequently observed in patients with perineural infiltration.

By contrast, Tonini et al. described that nuclear survivin overexpression in 67 resection specimens from patients with PDAC strongly correlated with longer postoperative survival, whereas cytoplasmic overexpression represented a negative prognostic marker [43].

Dong et al. recently analysed survivin expression in a series of 80 patients with PDAC. In their study, high protein expression levels of survivin in the primary tumours (81.0%) as well as elevated serum concentrations (61.5%) were significantly associated with perineural invasion, venous invasion, lymph node status, and independently correlated with overall survival [44]. Comparable findings in a series of 80 cases were presented by Ren et al. before also demonstrating significant correlations between high survivin serum levels and perineural and venous infiltration, lymph node metastasis, histologic grade, and primary tumour stage resulting in a shortened overall survival as an independent prognostic factor [45]. Lee et al. found survivin expression in 94% of 46 pancreatic carcinomas, which was also associated with perineural invasion and, beyond this, suggested a better responsiveness to chemotherapy [24].

Due to its significant correlation with the stage determining histopathologic parameters and the prognosis of PDAC patients, the quantification of serum survivin levels might serve for screening purposes potentially above the sensitivity and specificity of CA19-9 without being elevated in benign biliary obstruction [46]. In a recent study, Chang et al. demonstrated that exosomes in the serums of patients with PDAC harbouring KRAS mutations were carrying high amounts of survivin, as did KRAS-transformed fibroblasts and pancreatic cancer cells [47]. Moreover, survivin-enriched exosomes were able to increase the experimental survival of serum-deprived cells and interfere with the cells’ susceptibility to paclitaxel. Thus, survivin serum levels may not only serve for earlier detection of pancreatic adenocarcinoma but could also potentially predict their aggressiveness and responsiveness to systemic therapy.

In 2018, Zhou et al. matched samples of resected PDAC with non-malignant pancreatic tissue by TMA-based immunohistochemistry comprising 306 cases [48]. Other than in our series raising immunoreactivity scores (IRS), H scores were used to determine survivin overexpression [49]. Aside from regional lymph node status, vessel invasion, and adjuvant chemotherapy, the authors identified high nuclear survivin expression in the primary tumours as an additional independent prognostic factor due to significantly shorter disease-specific survival intervals. However, residual tumour load, perineural invasion, and UICC stage representing established markers in the assessment of pancreatic adenocarcinoma were not surveyed and included in the statistical analyses.

As in several study collectives before, we found that high patient age at the time of operation (also confirmed for most patient sets in the subgroup analyses), venous invasion (V1), and residual tumour burden after surgery at the resection site (R1) were independent prognostic factors for postoperative overall survival. Moreover, distant liver metastasis (M1) was significantly related with shortened survival intervals, while weak differentiation and strong cytoplasmic survivin expression at the invasion front were only marginally associated with limited overall survival.

Breaking down the patient cohort into clusters and submitting them to subgroup analyses, we delineated the presence of distant metastasis (M1) and positive resection margins (R1) as negative prognostic markers in PDAC of the pancreatic head and tail.

Since their growth usually does not result in painless icterus as a clinical key finding of tumours localised in the pancreatic head, adenocarcinomas of the pancreatic tail are mainly diagnosed serendipitously or by becoming symptomatic in late stage disease. However, no significant evidence exists so far that the localisation of primary PDAC in the pancreas reflects relevant biological differences with potential clinical implications for their treatment.

In PDAC of the pancreatic tail, lymphatic vessel invasion (L1) correlated with limited overall survival, whereas regional lymph node metastasis (N1) was of marginal prognostic relevance. As expected, the presence of distant metastasis (M1) represented an independent negative prognostic marker for overall survival in patients with adenocarcinoma of the pancreatic head.

We wish to emphasise that with multiple testing, accidental statistical significances may occur with increasing test runs and risk to be mistaken for falsely positive correlations. Given the limited number of patients included in our study, statistical adjustments for multiple testing were not to be advocated. Aware of these limitations, we discuss the results of our subgroup analyses with special caution realising that future studies with higher case numbers are needed to confirm and extend our findings. With careful interpretation of its prognostic relevance, high cytoplasmic expression levels at the invasion front statistically grouped with limited overall survival in cases without a residual tumour burden (R0), in the cluster of M0 patients, in patients staged both R0 and M0, as well as in patients with PDAC of the pancreatic tail. However, cytoplasmic expression of survivin failed to dominate as independent prognostic marker for overall survival in PDAC. In contrast, nuclear expression of survivin proved to be an independent prognostic biomarker for PDAC of the pancreatic tail in the multivariate calculations.

Assessing disease-free survival intervals, which were available in no more than 40% of the patients, residual tumour burden (R1) and otherwise nuclear overexpression of survivin in central tumour areas correlated independently with limited prognosis. Significance for elevated expression levels of survivin along the invasion front could be demonstrated only in the univariate analyses.

With their prognostic impact for PDAC patients, our findings support the implementation of molecular therapy approaches targeting survivin. Whilst several adjuvant chemotherapeutic regimes have been studied in large-scale prospective randomised trials improving postoperative survival intervals over time, the prognostic benefits for the patients remain unsatisfactory and novel therapeutic agents are desperately needed for patients with PDAC [6,50,51,52].

Already in 2005, Kami et al. demonstrated that silencing survivin mRNA expression in pancreatic cancer cell lines by siRNA reduced their radioresistency [53]. Comparably, the administration of antisense oligonucleotides to suppress the expression of survivin in pre-clinical applications resulted in a sensitisation of tumour cells to chemotherapeutic agents as paclitaxel and taxol [54,55].

The small molecule inhibitor of survivin YM155 (sepantronium bromide), on the other hand, suppresses the transactivation of survivin through direct binding to its promoter. YM155 has been examined in various cancer cell types, including melanomas and lymphomas, as well as prostate, lung, and breast cancers. Xenograft models have shown the antiproliferative efficacy of YM155 in combination with chemotherapy as well as in monotherapeutic approaches also in pancreatic cancer models [56,57,58].

The suppression of survivin by the inhibitor YM155 and its derivative termed UFSHR in tumour cell lines derived from human PDAC xenografts resulted in the reduction of cell proliferation and the induction of apoptosis [59]. Brown et al. demonstrated survivin expression in 68% of the primary tumours. In the same study from 2019, the authors extracted data from the TCGA database (The Cancer Genome Atlas; https://www.cancer.gov/about-nci/organization/ccg/research/structural-genomics/tcga, accessed on 15 July 2019), whereby in the analysis of 170 PDAC patients comparably high expression levels were related to significantly shorter mean survival intervals and survivin was statistically identified as an independent parameter correlating with overall survival. Moreover, survivin expression was detected in animal xenografts derived from human PDAC as well as in the cell lines having emerged from these. Applying two inhibitors—YM155 and UFSHR—to primary pancreatic cancer cell lines reduced their survivin expression and showed pro-apoptotic, anti-migratory, and anti-proliferative effects as expected.

The transcriptional and post-transcriptional regulation of the BIRC5 gene expression encoding for survivin are yet not fully understood. The BIRC5 promoter region contains binding sites for several transcription factors, which apparently interfere by competing for their partially overlapping binding sites. Among these, NF-κB, Sp1, Sp3, and Stat3 activate survivin gene promotion, whereas p53, APC, and Egr1 have been demonstrated to suppress BIRC5 expression [60,61]. In this context, YM155 treatment modified the cellular distribution of Sp1 suggesting that the therapeutic application of YM155 prevented Sp1 from docking to its designated promoter binding site to sustain survivin transcription [22]. While survivin is supposed to be regulated mainly at the transcriptional level, the epigenetic regulation of BIRC5 in PDAC remains to be elucidated. Whereas abnormal DNA methylation in promoter regions of oncogenes as well as in tumour suppressor genes can be observed in most cancers, both hypermethylation and hypomethylation in the promoter sequence of BIRC5 may correlate with survivin overexpression. Moreover, several studies demonstrated no significant differences in the methylation status of the BIRC5 promoter CpG island between healthy and malignant tissues. With regard to the permissive and regressive effects of post-translational histone modification, the assembly of a suppressive chromatin complex in the 5’-flanking promoter region of BIRC5 by epigenetic regulatory proteins resulted in nuclear gene repression while treated by doxorubicin [62,63]. The treatment of two PDAC cell lines (Panc-1, Capan2) with the histone-modifying molecule TSA targeting the histone deacetylases (HDAC) in combination with the Stat3 inhibiting silibinin showed an anti-proliferative effect that correlated with decreased survivin expression [64]. Before, the combined treatment of the gemcitabine-resistant PDAC cell lines Panc-1 and BxPC-3 with the HDAC inhibitor SAHA as an epigenetic modulator and the Smoothened antagonist SANT-1 inhibiting Hedgehog signalling resulted in the suppression of cell proliferation and induction of apoptosis, which again was related to the expression and nuclear distribution of survivin [65].

However, due to the different methylation status in different tissues with sometimes diametrically opposite effects of hyper- and hypomethylation on survivin expression, cancer-specific studies are needed to further investigate the underlying regulation of BIRC5 gene expression before existing epigenetic therapeutic approaches can find their way into clinical application in the treatment of PDAC patients with chemoresistant tumour burden.

Concerning promising therapy approaches directly addressing survivin, immunotherapeutic approaches comprising vaccine strategies based on T lymphocytes or dendritic cells (DC) are currently in progress. In the ongoing TACTOPS study (TAA Specific Cytotoxic T Lymphocytes in Patients with Pancreatic Cancer; Clinical Trials.gov Identifier: NCT03192462), patients with PDAC receive cytotoxic T lymphocytes directed against a panel of tumour-associated antigens (TAA) including survivin.

Recently, delimiting actionable genetic changes in the “Know Your Tumour” (KYT) programme by molecular profiling resulted in prolonged survival of metastasised PDAC patients after receiving customised therapy regimes with an increase from 1.51 years to 2.58 years of overall survival [66]. The clinical implementation of targeted therapy options should add to these encouraging results.

## 5. Conclusions

In summary, we investigated for the first time the expression of both cytoplasmic and nuclear survivin specifically in distinct tumour compartments of PDAC and demonstrated that omnipresent cytoplasmic and nuclear survivin expression in the tumour cells of primary PDAC and corresponding lymph node metastases were significantly higher in comparison to adjacent healthy pancreatic tissue. Furthermore, we demonstrated a correlation between the level of survivin expression in the central and marginal tumour areas and the clinicopathological variables critical for prognosis. Our investigations foster previous insights and improve the understanding that in PDAC the upregulation of both cytoplasmic and nuclear survivin expression point towards more aggressive tumour phenotypes with advanced disease stages that are comparatively often accompanied by synchronous distant metastasis. Given the prognostic impact of survivin overexpression in our PDAC patient collective and the availability of established inhibitors, our results argue for molecular and immunotherapeutic approaches targeting survivin that should be used in both monotherapeutic and combined clinical trials to improve the currently unsatisfactory overall disease outcome.

## Figures and Tables

**Table 1:** Patient characteristics (*n* = 236) ^1^.

Variables	No. of Patients (%)
Total	236
Age	
Median (range in years)	68 (41–95 yrs.)
Gender	
Male	126 (53.4%)
Female	110 (46.6%)
Tumour localisation	
Pancreatic head	217 (91.9%)
Pancreatic tail	19 (8.1%)
Primary tumour stage	
T1	1 (0.4%)
T2	11 (4.7%)
T3	214 (90.7%)
T4	10 (4.2%)
Regional lymph node metastasis	
N0	42 (17.8%)
N1	194 (82.2%)
Distant metastasis	
M0	203 (86.0%)
M1	33 (14.0%)
UICC Stage	
UICC IA	0
UICC IB	2 (0.8%)
UICC IIA	31 (13.1%)
UICC IIB	165 (69.9%)
UICC III	7 (3.0%)
UICC IV	31 (13.1%)
Grading	
G1/G2	125 (52.9%)
G3	110 (46.6%)
ND	1 (0.5%)
Perineural invasion	
Pn0	23 (9.7%)
Pn1	96 (40.7%)
ND	117 (49.6%)
Lymphatic vessel invasion	
L0	75 (31.8%)
L1	91 (31.8%)
ND	70 (29.7%)
Venous invasion	
V0	116 (49.1%)
V1	49 (20.8%)
ND	71 (30.1%)
Residual tumour	
R0	184 (78.0%)
R1	52 (22.0%)

^1^ ND = not determined; UICC = Union Internationale Contre le Cancer (Union for International Cancer Control, UICC).

**Table 2:** Nuclear survivin expression ^1^.

	Central tumour area			Tumour invasion front			Lymph node metastasis		
	Low (*n* = 125)	High (*n* = 95)	*p*-Value	Low (*n* = 92)	High (*n* = 97)	*p*-Value	Low (*n* = 51)	High (*n* = 97)	*p*-Value
Median age									
≤68 yrs.	72 (58.5%)	51 (41.5%)	0.586	50 (46.3%)	58 (53.7%)	0.466	29 (32.2%)	61 (67.8%)	0.484
>68 yrs.	53 (54.6%)	44 (45.4%)		42 (51.9%)	39 (48.1%)		22 (37.9%)	36 (62.1%)	
Gender									
male	57 (55.3%)	46 (44.7%)	0.685	45(45.5%)	54 (44.5%)	0.834	30 (38.0%)	49 (62.0%)	0.388
female	68 (58.1%)	49 (41.9%)		47(52.2%)	43 (47.8%)		21 (30.4%)	48 (69.6%)	
Primary tumour									
T1/T2	5 (50.0%)	5 (50.0%)	0.750	3 (30.0%)	7 (70.0%)	0.334	3 (37.5%)	5 (62.5%)	1.000
T3/T4	118 (56.7%)	90 (43.3%)		87 (49.2%)	90 (50.8%)		47 (33.8%)	92 (66.2%)	
Regional lymph node metastasis									
N0	23 (53.5%)	20 (46.5%)	0.732	18 (51.4%)	17 (48.6%)	0.852	3 (50.0%)	3 (50.0%)	0.415
N1	102 (57.6%)	75 (42.4%)		74 (48.1%)	80 (51.9%)		48 (33.8%)	94 (66.0%)	
Distant metastasis									
M0	111 (58.4%)	79 (41.6%)	0.240	83(51.6%)	78(48.4%)	0.067	44 (35.5%)	80 (64.5%)	0.643
M1	14 (46.7%)	16 (53.3%)		9(32.1%)	19(67.9%)		7 (29.2%)	17 (70.8%)	
UICC Stage									
I/II	107 (57.8%)	78 (42.2%)	0.577	79 (50.6%)	77 (49.4%)	0.257	44 (35.2%)	81 (64.8%)	0.812
III/IV	18 (51.4%)	17 (48.6%)		13 (39.4%)	20 (60.6%)		7 (30.4%)	16 (69.6%)	
Grading									
G2	58 (51.3%)	55 (48.7%)	0.076	44 (44.0%)	56 (56.0%)	0.242	27 (35.1%)	50 (64.9%)	1.000
G3	66 (63.5%)	38 (36.5%)		47 (53.4%)	41 (46.6%)		24 (34.8%)	45 (65.2%)	
Venous invasion									
V0	62 (57.9%)	45 (42.1%)	0.483	43 (45.7%)	51 (54.3%)	0.850	25 (34.7%)	47 (65.3%)	0.836
V1	24 (51.1%)	23 (48.9%)		17 (43.6%)	22 (56.4%)		15 (38.5%)	24 (61.5%)	
Lymphatic invasion									
L0	36 (52.9%)	32 (57.1%)	0.416	28 (47.5%)	31 (52.5%)	0.604	17 (40.5%)	25 (59.2%)	0.543
L1	53 (60.2%)	35 (39.8%)		32 (42.7%)	43 (57.3%)		23 (33.8%)	45 (66.2%)	
Perineural invasion									
Pn0	16 (72.7%)	6 (27.3%)	0.147	12 (66.7%)	6 (33.3%)	**0.035**	8 (61.5%)	5 (38.5%)	0.064
Pn1	46 (52.9%)	41 (47.1%)		29 (37.7%)	48 (62.3%)		23 (32.9%)	47 (67.1%)	
Residual tumour									
R0	95 (55.2%)	77 (44.8%)	0.412	76 (50.7%)	74 (39.3%)	0.369	38 (33.3%)	76 (66.7%)	0.682
R1	30 (62.5%)	18 (37.5%)		16 (41.0%)	23 (59.0%)		13 (38.2%)	21 (61.8%)	

^1^ UICC = Union Internationale Contre le Cancer (Union for International Cancer Control, UICC).

**Table 3:** Cytoplasmic survivin expression ^1^.

	Central tumour area			Tumour invasion front			Lymph node metastasis		
	Low (*n* = 127)	High (*n* = 93)	*p*-Value	Low (*n* = 97)	High (*n* = 92)	*p*-Value	Low (*n* = 63)	High (*n* = 85)	*p*-Value
Median age									
≤68 yrs.	67 (54.5%)	56 (45.5%)	0.336	49 (45.4%)	59 (54.6%)	0.077	40 (44.4%)	50 (55.6%)	0.612
>68 yrs.	60 (61.9%)	37 (38.1%)		48 (59.3%)	33 (40.7%)		23 (39.7%)	35 (60.3%)	
Gender									
male	68 (58.1%)	49 (41.9%)	1.000	47 (47.5%)	52 (52.5%)	0.309	33 (41.8%)	46 (58.2%)	0.869
female	59 (57.3%)	44 (42.7%)		50 (55.6%)	40 (44.4%)		30 (43.5%)	39 (56.5%)	
Primary tumour									
T1/T2	8 (80.0%)	2 (20.0%)	0.197	6 (60.0%)	4 (40.0%)	0.749	3 (37.5%)	5 (62.5%)	1.000
T3/T4	118 (56.7%)	90 (43.3%)		91 (51.4%)	86 (48.6%)		60 (43.2%)	79 (56.8%)	
Regional lymph node metastasis									
N0	23 (53.5%)	20 (46.5%)	0.606	14 (40.0%)	21 (60.0%)	0.189	3 (50.0%)	3 (50.0%)	0.700
N1	104 (58.8%)	73 (41.2%)		83 (53.9%)	71 (46.1%)		60 (42.3%)	82 (57.7%)	
Distant Metastasis									
M0	116 (61.1%)	74 (38.9%)	**0.016**	86 (53.4%)	75 (46.6%)	0.219	52 (41.9%)	72 (58.1%)	0.822
M1	11 (36.7%)	19 (63.3%)		11 (39.3%)	17 (60.7%)		11 (45.8%)	3 (54.2%)	
UICC Stage									
I/II	114 (61.6%)	71 (38.4%)	**0.009**	85 (54.5%)	71 (45.5%)	0.084	52 (41.6%)	73 (58.4%)	0.649
III/IV	13 (37.1%)	22 (62.9%)		12 (36.4%)	21 (63.6%)		11 (47.8%)	12 (52.2%)	
Grading									
G2	65 (57.5%)	48 (42.5%)	1.000	50 (50.0%)	50 (50.0%)	0.772	32 (41.6%)	45 (58.4%)	0.739
G3	60 (57.7%)	44 (42.3%)		46 (52.3%)	42 (47.7%)		31 (44.9%)	38 (55.1%)	
Venous invasion									
V0	60 (56.1%)	47 (43.9%)	1.000	41 (43.6%)	53 (56.4%)	**0.022**	26 (36.1%)	46 (63.9%)	0.683
V1	27 (57.4%)	20 (42.6%)		26 (66.7%)	13 (33.3%)		16 (41.0%)	23 (59.0%)	
Lymphatic invasion									
L0	39 (57.4%)	29 (42.6%)	1.000	28 (47.5%)	31 (52.5%)	0.731	18 (42.9%)	24 (57.1%)	0.552
L1	50 (56.8%)	38 (43.2%)		38 (50.7%)	37 (49.3%)		25 (36.8%)	43 (63.2%)	
Perineural invasion									
Pn0	15 (68.2%)	7 (31.8%)	0.337	11 (61.1%)	7 (38.9%)	0.306	6 (46.2%)	7 (53.8%)	1.000
Pn1	48 (55.2%)	39 (44.8%)		36 (46.8%)	41 (53. 2%)		30 (42.9%)	40 (57.1%)	
Residual tumour									
R0	100 (58.1%)	72 (41.9%)	0.869	76 (50.7%)	74 (49.3%)	0.858	45 (39.5%)	69 (60.5%)	0.173
R1	27 (56.3%)	21 (43.8%)		21 (53.8%)	18 (46.2%)		18 (52.9%)	16 (47.1%)	

^1^ UICC = Union Internationale Contre le Cancer (Union for International Cancer Control, UICC).

**Table 4:** Overall survival analysis ^1^.

Variable	Univariate analysis	Multivariate analysis		
	*p*-Value	HR	95% CI	*p*-Value
Age (median 68 yrs.)	**0.009**	**1.737**	**1.121–2.692**	**0.013**
Male vs. female	0.490	NS	NS	NS
T1/2 vs. T3/4	0.432	NS	NS	NS
N0 vs. N1	0.384	NS	NS	NS
M0 vs. M1	**<0.001**	NS	NS	NS
G1/2 vs. G3	0.071	NS	NS	NS
Pn0 vs. Pn1	0.427	NS	NS	NS
L0 vs. L1	0.794	NS	NS	NS
V0 vs. V1	**0.021**	**1.615**	**1.024–2.546**	**0.039**
R0 vs. R1	**0.011**	**2.421**	**1.391–4.216**	**0.002**
High vs. low nuclear survivin (central tumour area)	0.769	NS	NS	NS
High vs. low nuclear survivin (invasion front)	0.793	NS	NS	NS
High vs. low cytoplasmic survivin (central tumour area)	0.782	NS	NS	NS
High vs. low cytoplasmic survivin (invasion front)	0.072	NS	NS	NS

^1^ HR = hazard ratio; 95% CI = 95% confidence interval; NS: not significant.

**Table 5:** Univariate subgroup analysis for overall survival ^1^.

Variable	Patient subgroup				
	R0 (*n* = 184)	R0 + M0 (*n* = 168)	M0 (*n* = 203)	Pancreatic head PDAC (*n* = 217)	Pancreatic tail PDAC (*n* = 19)
Age (median 68 yrs.)	**0.024**	**0.005**	**0.002**	**0.024**	0.480
Male vs. female	0.944	0.996	0.677	0.457	0.830
T1/2 vs. T3/4	0.561	000.511	0.647	0.682	0.480
UICC I/II vs. UICC III/IV ^2^	0.561	0.499	0.113	0.095	0.397
N0 vs. N1	0.950	0.601	0.651	0.203	0.074
M0 vs. M1	0.308	—	—	**0.001**	0.074
G1/2 vs. G3	0.939	0.775	0.183	0.052	0.818
Pn0 vs. Pn1	0.657	0.691	0.557	0.814	0.522
L0 vs. L1	0.374	0.207	0.462	0.819	**0.046**
V0 vs. V1	0.605	0.713	0.093	**0.041**	0.370
R0 vs. R1	—	—	0.347	0.056	**0.047**
High vs. low nuclear survivin (central tumour area)	0.645	0.501	0.782	0.747	0.317
High vs. low nuclear survivin (invasion front)	0.819	0.863	0.943	0.992	0.363
High vs. low cytoplasmic survivin (central tumour area)	0.573	0.652	0.398	0.745	0.841
High vs. low cytoplasmic survivin (invasion front)	**0.025**	**0.029**	**0.037**	0.481	**0.022**

^1^ Results presented as p-values. ^2^ UICC I/II vs. III/IV—grouped tumour stage classifications according to the Union Internationale Contre le Cancer (Union for International Cancer Control).

**Table 6:** Multivariate subgroup analysis for overall survival ^1^.

Variable	Patient subgroup				
	R0 (*n* = 184)	R0 + M0 (*n* = 168)	M0 (*n* = 203)	Pancreatic head PDAC (*n* = 217)	Pancreatic tail PDAC (*n* = 19)
Age (median 68 yrs.)	NS	**1.5** (**1.0–2.2**)	**1.5** (**1.1–2.1**)	NS	NS
Male vs. female	NS	NS	NS	NS	NS
T1/2 vs. T3/4	NS	NS	NS	NS	NS
UICC I/II vs. UICC III/IV ^2^	NS	NS	NS	NS	NS
N0 vs. N1	NS	NS	NS	NS	NS
M0 vs. M1	NS	—	—	**2.1** (**1.3–3.3**)	NS
G1/2 vs. G3	NS	NS	NS	NS	NS
Pn0 vs. Pn1	NS	NS	NS	NS	NS
L0 vs. L1	NS	NS	NS	NS	NS
V0 vs. V1	NS	NS	NS	NS	NS
R0 vs. R1	—	—	NS	NS	NS
High vs. low nuclear survivin (central tumour area)	NS	NS	NS	NS	**13.5** (**1.4–129.7**)
High vs. low nuclear survivin (invasion front)	NS	NS	NS	NS	NS
High vs. low cytoplasmic survivin (central tumour area)	NS	NS	NS	NS	NS
High vs. low cytoplasmic survivin (invasion front)	NS	NS	NS	NS	NS

^1^ Results presented as HR (hazard ratios) followed by 95% CI (confidence interval) in parentheses; NS: not significant. ^2^ UICC I/II vs. III/IV—grouped tumour stage classifications according to the Union Internationale Contre le Cancer (Union for International Cancer Control).

**Table 7:** Disease-free survival analysis ^1^.

Variable	Univariate analyses	Multivariate analyses		
	*p*-Value	HR	95% CI	*p*-Value
Age (median 68 yrs.)	0.481	NS	NS	NS
Male vs. female	0.498	NS	NS	NS
T1/2 vs. T3/4	0.551	NS	NS	NS
N0 vs. N1	0.621	NS	NS	NS
M0 vs. M1	0.552	NS	NS	NS
G1/2 vs. G3	0.481	NS	NS	NS
Pn0 vs. Pn1	0.219	NS	NS	NS
L0 vs. L1	0.634	NS	NS	NS
V0 vs. V1	0.207	NS	NS	NS
R0 vs. R1	**0.043**	**1.885**	**1.065-3.161**	**0.029**
Nuclear survivin (central tumour area)	**0.024**	**1.798**	**1.037-3.118**	**0.012**
Nuclear survivin (invasion front)	**0.050**	NS	NS	NS
Cytoplasmic survivin (central tumour area)	0.362	NS	NS	NS
Cytoplasmic survivin (invasion front)	0.416	NS	NS	NS

^1^ HR = hazard ratio; 95% CI = 95% confidence interval; NS: not significant.

## Data Availability

The data presented in this study are available on request from the corresponding author.
